# Absent right pulmonary artery with patent ductus arteriosus and Eisenmenger syndrome

**DOI:** 10.1093/ehjimp/qyag169

**Published:** 2026-09-30

**Authors:** José Martín Alanís-Naranjo, Regina de la Mora-Cervantes, Cielmar Galeana-Abarca, Ana María Rosas-Vázquez

**Affiliations:** Cardiovascular Imaging Department, Instituto Nacional de Cardiología Ignacio Chávez, Mexico City, Mexico; Cardiovascular Imaging Department, Instituto Nacional de Cardiología Ignacio Chávez, Mexico City, Mexico; Cardiovascular Imaging Unit, Hospital Regional 1° de Octubre, Instituto de Seguridad y Servicios Sociales de los Trabajadores del Estado (ISSSTE), Mexico City, Mexico; Cardiovascular Imaging Department, Instituto Nacional de Cardiología Ignacio Chávez, Mexico City, Mexico

**Keywords:** Cardiac computed tomography, Congenital heart disease, Unilateral absence of the pulmonary artery, Patent ductus arteriosus, Eisenmenger syndrome, Pregnancy

A 23-year-old woman was referred for evaluation of persistent oxygen desaturation (SpO_2_ 88%), incidentally identified during hospitalisation for the second delivery. Exertional dyspnoea and palpitations had been present since childhood, precluding participation in sports or school physical education, but residence in a remote area with limited access to specialist care had prevented further evaluation. Both pregnancies, the first at age 21, were uncomplicated and resulted in term vaginal deliveries, with no symptom worsening following the second delivery. Physical examination revealed a continuous murmur and digital clubbing of the lower limbs. Laboratory tests showed erythrocytosis (haemoglobin 19 g/dL).

Echocardiography showed right heart dilatation, preserved biventricular systolic function (left ventricular ejection fraction [LVEF] 66%; tricuspid annular plane systolic excursion [TAPSE] 18 mm), severe tricuspid regurgitation, and an estimated pulmonary artery systolic pressure of 113 mmHg, with Doppler evidence of right-to-left flow through a poorly visualised patent ductus arteriosus (PDA).

Cardiac computed tomography (CCT) confirmed right heart dilatation and the PDA, while revealing absence of the right pulmonary artery and a systemic-pulmonary collateral supplying the right lung (*Figure 1*, Supplementary data online, *Video S1*). Right heart catheterisation confirmed severe pulmonary hypertension (mean pulmonary artery pressure 76 mmHg, pulmonary vascular resistance 25 WU) with a right-to-left shunt through the PDA (Qp/Qs 0.79), consistent with Eisenmenger physiology.

**Figure 1:**
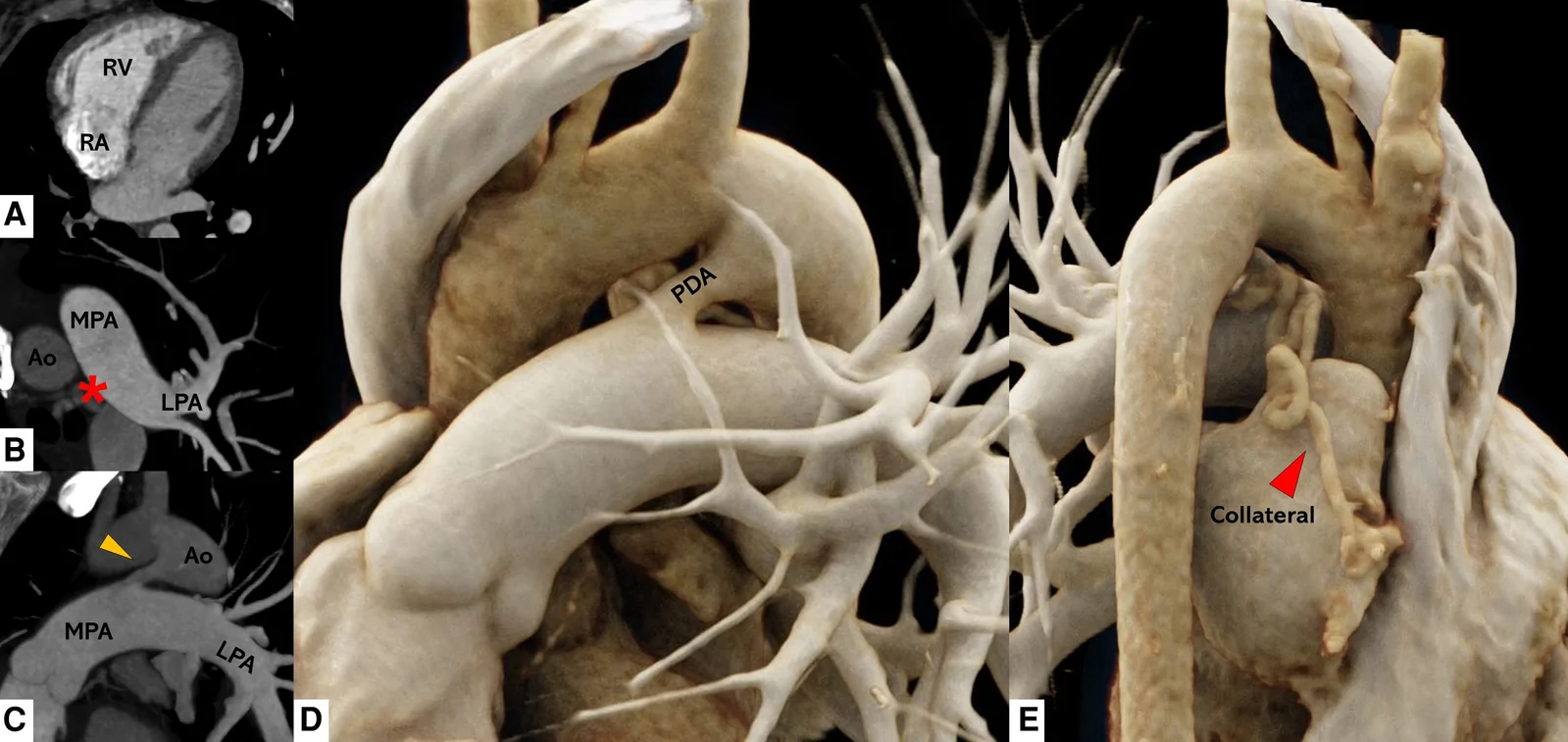
Cardiac computed tomography angiography. Right heart dilatation, absence of the right pulmonary artery (asterisk), a PDA , and a systemic-pulmonary collateral. (*A*) Axial MPR four-chamber view. (*B*) Axial MIP at the level of the main and left pulmonary arteries. (*C*) Oblique MIP depicting the PDA connecting the descending aorta to the left pulmonary artery (arrowhead). (*D*) Cinematic 3D VRT reconstruction highlighting the PDA. (*E*) Cinematic 3D VRT reconstruction demonstrating the systemic-pulmonary collateral arising from the aortic arch and supplying the right lung (arrowhead). Ao, aorta; LPA, left pulmonary artery; MIP, maximum intensity projection; MPA, main pulmonary artery; MPR, multiplanar reconstruction; PDA, patent ductus arteriosus; RA, right atrium; RV, right ventricle; VRT, volume rendering technique.

The anatomical and haemodynamic findings precluded PDA closure. Although heart–lung transplantation was considered, socioeconomic limitations affecting adherence to post-transplant immunosuppression favoured pulmonary hypertension-targeted therapy with sildenafil and bosentan, alongside counselling on pregnancy-related risks and avoidance of future pregnancy.

The patient remained in WHO functional class II without hospitalisations throughout 9 years of follow-up; current functional assessment showed a 6-minute walk distance of 478 m, with no symptom progression, syncope, or signs of right heart failure.

This case provides further insight into the clinical course of this rare congenital heart disease during pregnancy and delivery, both of which remained uncomplicated despite Eisenmenger physiology, and into long-term survival on medical therapy. Integration of CCT findings with invasive haemodynamics provided the anatomical and haemodynamic basis for therapeutic decision-making.

## Supplementary Material

qyag169_Supplementary_Data

## Data Availability

The data underlying this article are available within the article and its supplementary materials.

## Lead author biography

Martín Alanís-Naranjo received his medical degree from the Universidad Michoacana de San Nicolás de Hidalgoin Morelia, Mexico. Following postgraduate training in Internal Medicine and Cardiology, he completed advanced training in cardiovascular magnetic resonance and cardiac computed tomography at the Instituto Nacional de Cardiología Ignacio Chávez in Mexico City, Mexico. He is currently pursuing a master’s degree in Cardiac Imaging at Hospital Clínico San Carlos in Madrid, Spain.

